# ^18^F-Fluoromisonidazole in tumor hypoxia imaging

**DOI:** 10.18632/oncotarget.21662

**Published:** 2017-10-07

**Authors:** Zuoyu Xu, Xiao-Feng Li, Hongyan Zou, Xilin Sun, Baozhong Shen

**Affiliations:** ^1^ Molecular Imaging Research Center (MIRC), Harbin Medical University, Harbin, Heilongjiang, China; ^2^ TOF-PET/CT/MR Center, The Fourth Hospital of Harbin Medical University, Harbin, Heilongjiang, China

**Keywords:** hypoxia, ^18^F-Fluoromisonidazole, PET, tumor

## Abstract

Hypoxia is a common feature of solid tumors that is closely associated with radiotherapy and chemotherapy resistance, metastasis and tumors prognosis. Thus, it is important to assess hypoxia in tumors for estimating prognosis and selecting appropriate treatment procedures. ^18^F-Fluoromisonidazole positron emission tomography (^18^F-FMISO PET) has been widely used to visualize tumor hypoxia in a comprehensive and noninvasive way, both in the clinical and preclinical settings. Here we review the concept, mechanisms and detection methods of tumor hypoxia. Furthermore, we discuss the correlation between ^18^F-FMISO PET and other detection methods, current applications of ^18^F-FMISO PET and the development prospects of this imaging technology.

## INTRODUCTION

Hypoxia is a common feature of solid tumors. Despite its heterogeneity, it is tightly associated with tumor tissue survival and glucose metabolic pathways activity [1]. Hypoxia promotes angiogenesis, cancer invasiveness and resistance to radiotherapy and chemotherapy [2]. Furthermore, metastasis and prognosis have a close relationship with hypoxia [3]. Hypoxia can be classified into acute or chronic type. Acute hypoxia occurs when the microvasculatures surrounding tumor tissues close up, which is usually transient. And when the closed blood vessels reopen, the hypoxic cells are reoxygenated. In contrast, chronic hypoxia occurs when the tumor cells are distant from functional capillaries. It is typically irreversible or long-term. Chronic hypoxic cells may become necrotic or reoxygenated, or convert into more aggressive cell types. Several processes cause chronic hypoxia in cancer cells [4]. First, the increased oxygen demand from abnormal tumor cell proliferation may result in insufficient blood supply from the microvasculatures surrounding the tumor. Second, due to the gradual enlargement of the tumor, the cancer cells distance themselves from functional blood vessels. Finally, defects in tumor angiogenesis and abnormalities of microvascular function may cause a unstable oxygen environment [5]. Tumor hypoxia is spatially and temporally heterogeneous [6, 7]. Noninvasive methods for visualizing hypoxic imaging probes are necessary to detect the distribution of hypoxic cancer cells in the body. ^18^F-Fluoromisonidazole (^18^F-FMISO) is the most extensively used radiolabeled imaging tracer for hypoxia among several others. In this review, we introduce ^18^F-FMISO imaging with current hypoxia detection methods and applications in tumor hypoxia. Then discuss its advantages and disadvantages when compared with other hypoxia radiolabeled imaging tracers. Finally, we explore the potential development prospects of this imaging technology.

## SYNTHESIS OF ^18^F-FMISO AND BIODISTRIBUTION

**Figure 1:**
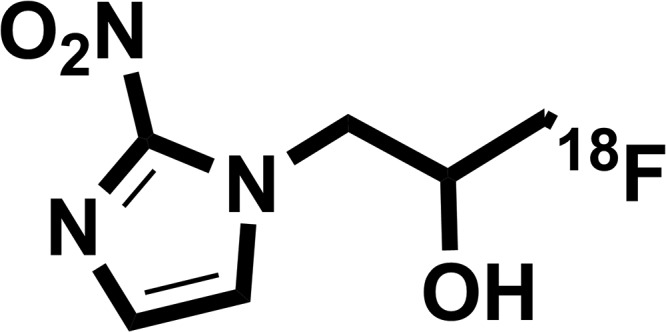
Chemical structure of ^18^F-FMISO

**Figure 2:**
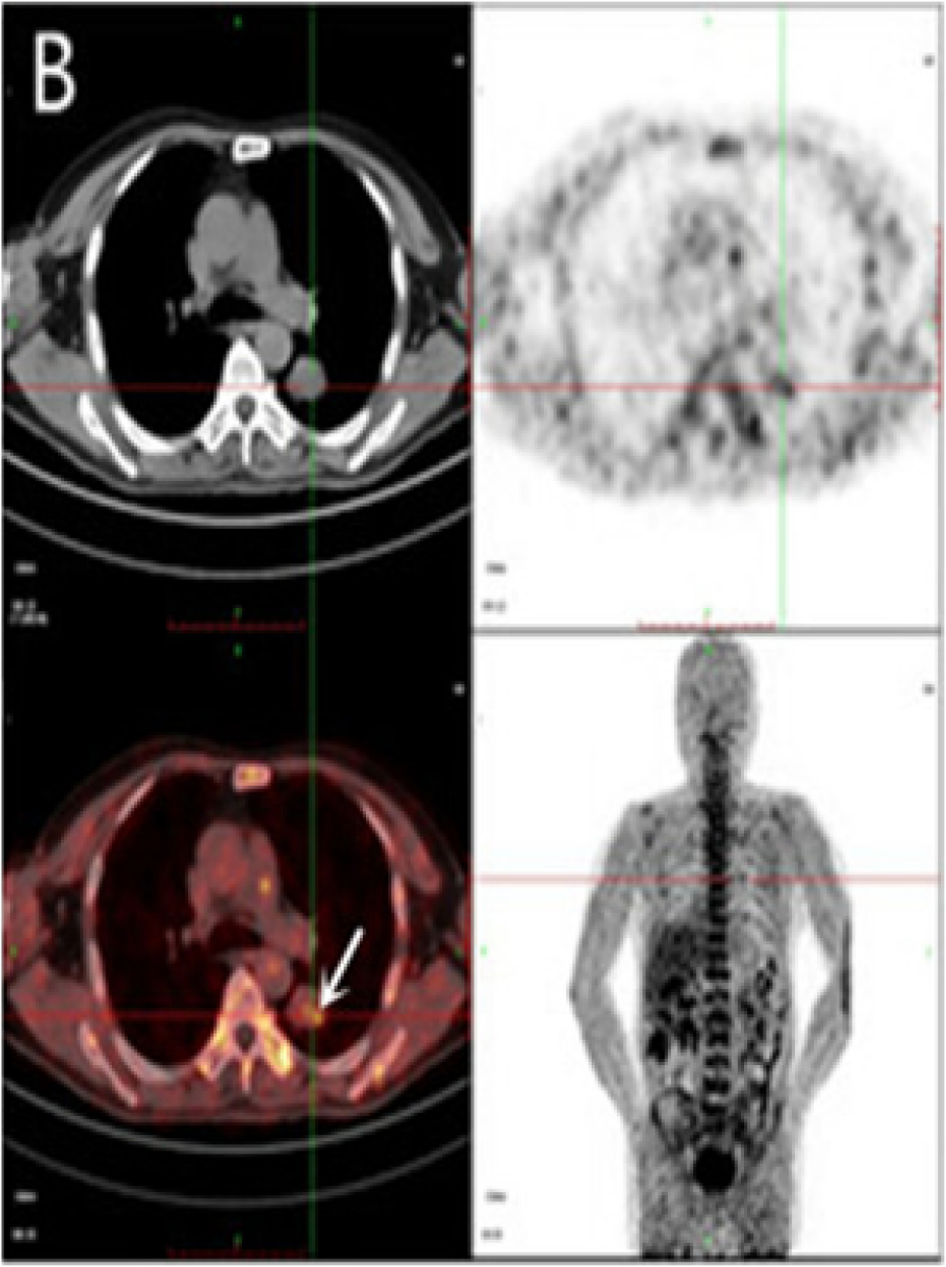
PET imaging Major organs and regions uptake at 2 h after injection of ^18^F-FMISO (**B**) in small cell lung cancer patient. Arrows point to tumor. (Wei Y, Zhao W, Huang Y, Yu Q, Zhu S, et al. (2016). A Comparative Study of Noninvasive Hypoxia Imaging with ^18^F-Fluoroerythronitroimidazole and ^18^F-Fluoromisonidazole PET/CT in Patients with Lung Cancer. PLOS ONE 11(6): e0157606. https://doi.org/10.1371/journal.pone.0157606).

FMISO has the chemical structure of 1-(2’nitro-1’-imidazolyl)-3-fluoro-2-propranol and when radiolabeled with fluorine-18, it becomes ^18^F-FMISO (Figure 1). ^18^F-FMISO is typically synthesized from a commercially available precursor as previously described [8] and purified by preparative high-pressure liquid chromatography. The final product has purity between 95% and 99%, specific activity between 1 and 3 Ci/mol, and pH between 5 and 8. The misonidazole biological half-life is 50 minutes. ^18^F-FMISO is metabolized by the liver and then excreted by the kidney and bladder. The lowest activity is noted in the blood, spleen, heart, lung, muscle, bone, and brain [9]. The biodistribution data in human subjects with lung cancer is demonstrated in Figure 2 [10].

## HYPOXIA DETECTION METHODS

Polarographic oxygen electrodes, which measure tissue oxygen pressure directly, are considered to be the gold standard for detecting tumor hypoxia. Oxygen partial pressure (PO_2_) of normal subcutaneous tissue is approximately 40–60 mmHg. The median PO_2_ of tumor tissue is about 10 mmHg. Hypoxia is defined as PO_2_ less than 10mmHg [11]. It is also possible to measure hypoxia indirectly in tissue sections by assessing protein expression using immunohistochemical methods such as endogenous cell markers and exogenous nitroimidazole-based assays [12]. Hypoxia inducible factor 1 (HIF-1, consisting of HIF-1α and HIF-1β) is an endogenous cell marker that regulates many genes, including vascular endothelial growth factor (VEGF) [13]. HIF-1 plays a central role in tumor progression. Exogenous hypoxic agents are mostly imidazole compounds such as pimonidazole and EF-5. Imaging methods of assessing for tumor hypoxia include MRI and PET.

Positron emission tomography (PET) is a widespread imaging technology that uses radioactive tracers to noninvasively detect tumors and monitor cancer therapy effectiveness [14]. ^18^F-FMISO is a nitroimidazole compound, which is labeled with radionuclides. After nitroimidazole enters the cell via the blood flow, it can occur redox reactions under the action of xanthine oxidase. In normal cells which are not hypoxia, reducted nitro group can be oxidized into the original substance by O_2_. While in hypoxic cells, reducted nitro group can’t be oxidized. It will stably combine with some cellular components permanently. ^18^F-FMISO can be detected by PET that has been widely used for tumor hypoxia imaging (Table 1). This is the first hypoxia PET tracer used in clinical settings, and currently there are some new tracers available, most derived from the family of 2-nitroimidazoles.

**Table 1:** Summary of studies using 18F-FMISO PET in tumor hypoxia

Tumor	Time points	Conclusion	Reference
HNSCC	120 min	Summarizing the ^18^F-FMISO uptake represents a global value for macroscopic tumor parts. As a noninvasive measurement this method seems highly feasible to evaluate the state of oxygenation in subjacent tumors.	[15]
STS	1.5 h, 2.5 h, 3.5 h	^18^F-FMISO PET in our setting seemed not to be feasible for the detection of tumour hypoxia in human soft tissue tumors.	[16]
OSCC	4 h	^18^F-FMISO uptake in the primary site of OSCC indicates a hypoxic environment with HIF-1α expression.	[19]
Glioma	120 min	The IRS of HIF-1α in the tumour did not correlate with the SUVtumor of ^18^F-FMISO in either newly diagnosed or recurrent glioma.	[23]
Glioma	120 min	Preoperative ^18^F-FMISO uptake is significantly correlated with the expression of VEGF in the tumour and might be used as a biomarker of antiangiogenic treatment in newly diagnosed malignant gliomas.	[23]
HNSCC	4 h	The values for ^18^F-FMISO PET uptake and hypoxic volume in head and neck tumors between the 2 ^18^F-FMISO scans were highly reproducible.	[31]
RCC	2 h	Although ^18^F-FMISO scans showed significant uptake in other solid tumors, there was only mild ^18^F-FMISO uptake in the present RCCs. The invasive measurements indicated that there was hypoxia in RCC, but the median PO2 did not fall below 9.5 mmHg.	[32]
HNSCC	162 min	Of 13 patients, 6 had well-correlated intratumor distributions of ^18^F-FMISO suggestive of chronic hypoxia.	[33]
FaDu and CAL-33 xenografted tumors	2 h	Whether the Wang model can be used to predict radiation response after serial ^18^F-FMISO PET imaging, needs to be confirmed in experimental and clinical studies.	[34]
Glioma	4 h	^18^F-FMISO PET may distinguish glioblastoma multiforme from lower grade gliomas.	[37]
Glioma	120 min	^18^F-FMISO PET is a potential tracer in the assessment of noninvasive tumor grading in newly diagnosed gliomas.	[38]
HNSCC	2 h	Hypoxia-targeted radiotherapy dose painting for head and neck cancer using FMISO PET is technically feasible, increases the TCP without increasing the NTCP, and increases the UTCP. ^18^F-FMISO PET is superior to uniform dose escalation.	[45]
C6 and 9L glioblastomas rat tumor model	dynamic ^18^F-FMISO PET (for a total of 2 hours)	This has implications for improved patient selection, particularly in clinical trials, for treatment with hypoxia-activated cytotoxic prodrugs, such as evofosfamide.	[49]
HNSCC	2 h	Hypoxia on ^18^F-FMISO PET imaging, in patients receiving a nontirapazamine-containing chemoradiotherapy regimen, is associated with a high risk of LRF.	[50]
A549 peritoneal micrometastases model	2 h	Microscopic non-small cell lung cancer metastases are severely hypoxic. ^18^F-FMISO PET is capable to image hypoxia noninvasively not only in macroscopic tumors but also in micrometastases growing in mice. ^18^F-misonidazole may be a promising agent to detect the burden of micrometastatic diseases.	[54]
glioblastoma multiforme	120 to 140 min	This type of imaging could be integrated into new treatment strategies to target hypoxia more aggressively in glioblastoma multiforme and could be applied to assess the treatment outcomes.	[22]
ER-α-positive breast cancer	2 and 4 h	^18^F-FMISO PET/CT can be used to predict primary endocrine resistance in ER-positive breast cancer.	[56]
mRCC	2–4 h	Sunitinib reduced hypoxia in initially hypoxic RECIST target metastases but did not induce significant hypoxia in nonhypoxic RECIST target metastases. Patients with initially hypoxic targets have shorter PFS than others.	[59]

HNSCC, head and neck squamous cell carcinoma; STS, soft tissue sarcomas; OSCC, oral squamous cell carcinoma; IRS, immunoreactivity score; RCC, renal cell carcinoma; TCP, tumor control probability; NTCP, normal tissue complication probability; UTCP, uncomplicated tumor control probability; LRF, locoregional failure; ER+, positive estrogen receptor; PFS, progression-free survival; RECIST, response evaluation criteria in solid tumors.

### ^18^F-FMISO PET vs. polarographic oxygen electrodes

Although polarographic oxygen electrodes are commonly used to measure hypoxia, this method presents several disadvantages. For instance, besides being invasive, polarographic oxygen electrodes can fail to detect the whole regions because of the known heterogeneity in tumor. In addition, the results of these measurements cannot be used for radiotherapy treatment planning. Gagel *et al.* reported there was a significant correlation between tumor-to-muscle ratio of ^18^F-FMISO and parameters of hypoxic fraction in head and neck cancer [15]. This indicated ^18^F-FMISO uptake represented a noninvasive measurement for macroscopic tumor hypoxia and seemed highly feasible to evaluate the state of oxygenation in tumors. To the opposite, a few studies showed no correlation between polarographic measurements and ^18^F-FMISO. Bentzen *et al* suggested ^18^F-FMISO PET seemed not to be feasible for the detection of tumor hypoxia in human soft tissue sarcomas [16]. Neither did it reflect the extent of hypoxia as determined with the oxygen electrode measurements. Mortensen *et al* analyzed the relationship between ^18^F-FMISO PET and Eppendorf electrode measurements by use of a virtual voxel model [17]. However, no correlation was observed, and in generally, tumors were more hypoxic based on Eppendorf measurements as compared to ^18^F-FMISO PET. One of possible explanations for the contrast results is polarographic electrodes can´t discriminate between hypoxia and necrosis, as both regions present low oxygen partial pressure. It may over estimate hypoxia. This may explain the diverging results of ^18^F-FMISO PET hypoxia imaging and polarographic measurements.

### ^18^F-FMISO PET vs. immunohistochemical findings

Endogenous cell markers and exogenous nitroimidazole-based assays have similar disadvantages with polarographic oxygen electrodes. One of the main early cellular events evoked by hypoxia is induction of HIF-1. HIF-1 induces the transcription of a wide variety of genes, including glycolysis, angiogenesis, survival pathways and invasion [18]. There are a few studies exploring the correlation between ^18^F-FMISO uptake and immunohistochemical expressions. Sato *et al.* demonstrated ^18^F-FMISO PET uptake was correlated with HIF-1α expression in oral squamous cell carcinoma [19]. Similarly, Norikane *et al* found the values of ^18^F-FMISO hypoxic volume showed a weak correlation with HIF-1α obtained on immunohistochemical examinations in head and neck cancer [20]. But in clinical gliomas researches, none of studies was able to show a correlation between ^18^F-FMISO uptake and expression of HIF-1α [21–23]. Except for the small sample size, there are several possibilities to explain the discrepancy between ^18^F-FMISO uptake and HIF-1α expression. For one thing, even though hypoxia is the main factor in activating HIF-1α, many non-hypoxic stimuli are also very easy to turn on this transcription factor, such as growth factors, cytokines, etc [24]. For another, HIF-1α expression may depend on the degree and duration of intermittent hypoxia in tumor [23]. Although hypoxia can increase HIF-1α protein expression, HIF-1α has a short half-life under normoxic conditions and the tissue becomes anoxic between resection and embedding [25, 26].

HIF-1 is thought to be key for up-regulating factors such as VEGF. Most of ^18^F-FMISO analysis of individual whole tumor volumes suggests no association between VEGF expression and hypoxia [27]. Heterogeneity in VEGF expression in individual tumors is likely a reflection of similar heterogeneity in tumor hypoxia. And VEGF expression in response to hypoxia is a local phenomenon rather than generalized in a tumor [27]. In recent years, Kawai *et al* investigated whether hypoxia detected by ^18^F-FMISO PET could accurately reflect biomarkers or prognostic factors treated by bevacizumab which is used as anti-angiogenic treatment [23]. The results revealed a weak correlation between the immunoreactivity score of VEGF and ^18^F-FMISO maximum standardized uptake value (SUV_max_) in newly diagnosed glioma brain tumors, but not in recurrent gliomas.

### ^18^F-FMISO PET vs. BOLD fMRI

BOLD fMRI (blood oxygenation-level dependent functional magnetic resonance imaging) technology utilizes the impact of the oxyhemoglobin and deoxyhemoglobin ratio changes onto the magnetic resonance signals to indirectly infer the state of PO_2_. Compared with ^18^F-FMISO imaging, it does not require to inject a compound. And, BOLD has a good temporal and spatial resolution, that can improve quality and contrast of images. Research has shown that hypoxia detection using BOLD fMRI shows a good correlation with PO_2_ inside tumors [28]. But BOLD fMRI is a semi-quantitative method, and affected by respiratory that is not suitable for respiratory system, such as lung cancer.

## APPLICATION OF ^18^F-FMISO PET

In addition to ^18^F-FMISO with other detection methods studies, there have been many settings using ^18^F-FMISO as a noninvasive examinations. Most of them are clinical settings. As a clinical agent, it has a good reproducibility so that can detect hypoxia, guide therapy, manage therapeutic efficacy and estimate prognosis. And also it is used in some preclinical cases to help further clinical studies.

### Detecting hypoxia

According to the research, the accuracy of malignant tumor detection in the lung using traditional ^18^F-FDG PET is more than 90% [29]. However, a significant proportion will be false positive results because ^18^F-FDG is a metabolic imaging tracer. Glycolysis is increased not only in tumor, but also in other diseases including granuloma, sarcoidosis, tuberculosis and inflammation [30]. So, it is difficult to distinguish atelectasis and local infections in lung tumors using ^18^F-FDG. Maybe the use of ^18^F-FMISO alone or in combination with ^18^F-FDG can improve the accuracy of diagnosis, especially in lung cancer. Furthermore, it has a good reproducibility. Okamoto *et al* addressed the reproducibility of ^18^F-FMISO PET uptake to detect tumor hypoxia by calculating SUV_max_, tumor/blood ratio and tumor/muscle ratio at two different time points in head and neck squamous cell carcinoma (HNSCC) patients [31]. The results confirmed the important role of ^18^F-FMISO in precisely and reproducibly in detecting tumor hypoxia. Although ^18^F-FMISO scans showed significant uptake in sarcomas, gliomas, head and neck tumors, there was only mild uptake in renal cell carcinoma (RCC). Lawrentschuk *et al* studied 7 of 17 patients with clear RCC [32]. The difference between tumor uptake and normal uptake in the contralateral kidney was not statistically significant. Except for the limited cases on hypoxia studies in RCC, one possible reason may be RCC was not as hypoxic a tumor as suspected. The application of ^18^F-FMISO in RCC is still to be determined.

Chronic and acute hypoxia are caused by oxygen diffusion and perfusion limitation, respectively. It is important to estimate the fraction of hypoxia in solid tumors because it plays an important role in tumor aggression, progression and metastasis. A study by Nehmeh *et al* attempted to detect areas of chronic hypoxia in HNSCC by comparing ^18^F-FDG scans taken at day 0 with ^18^F-FMISO scans at two different time points (day 1 and 4) [33]. The outline of the tumors was set with ^18^F-FDG as the threshold, and the outline of the hypoxia areas was set by ^18^F-FMISO in tumor/blood ratio ≥1.2 as the threshold. In 6 of 13 patients, the hypoxia areas detected by ^18^F-FMISO overlapped well, suggesting they corresponded to regions of chronic hypoxia. However, the data cannot identify regions of acute hypoxia. In a study by Maftei *et al*, serial ^18^F-FMISO PET/CT imaging was used in combination with immunofluorescence stainings (pimonidazole, CD31 and Hoechst 33342) of cryosections to assess the fraction of acute hypoxia in HNSCC tumors [34]. But no correlation between the results obtained by each method was found. Warren *et al* presented a computational model to simulate the effects of temporally-incoherent cyclic variations in perfusion on ^18^F-FMISO contrast [35]. Results suggested that the signal observed at late time points did not specifically relate to ‘chronic’ or ‘acute’ hypoxia. But it was representative of the time-averaged oxygenation during the imaging study.

### Grading

Glioma is one of the most common types of malignant primary brain tumors. Glioma has a variety of different grades, however, biopsies are not reliable for grading due to sampling errors resulting from tumor tissue heterogeneity. Errors in glioma grading effect treatment strategy and prognosis and can therefore have serious consequences. Since ^18^F-FMISO PET was first used in clinical glioma imaging by Valk *et al*, its advantages were immediately recognized and subsequently ^18^F-FMISO PET was introduced into glioma researches [36]. Necrotic tissues are not observed in grade III or lower grade gliomas. ^18^F-FMISO cannot detect necrosis, but necrosis even microscopic may be surrounded by a hypoxic area. That is sufficiently large to be identified by ^18^F-FMISO PET. Hirata *et al* found that ^18^F-FMISO uptake occurred only in glioblastoma multiforme (grade IV), and there was no significant uptake in lower gliomas (below grade IV) [37]. In this study, the sensitivity of both ^18^F-FMISO and ^18^F-FDG was 100%, but the specificity of the former was significantly higher (100%) than that of the latter (66%). Consistent with these findings, Cher *et al* and Yamamoto *et al* reported that grade IV glioma accumulated more ^18^F-FMISO than lower grade gliomas [21, 38]. Others have discussed these results and emphasized the importance of the imaging time for grading gliomas: 4 hours of imaging showed only the hypoxic zone; but 2 hours of imaging could reflect changes in blood flow with or without hypoxia [39, 40]. These studies show that ^18^F-FMISO PET may be useful not only for diagnosis, but also for grading and estimating prognosis of glioma.

### Guiding dose painting

HNSCC accounts for about 5% of all malignancies. Radiotherapy is currently one of the most effective treatments for HNSCC [41, 42]. However, radiation resistance is an inevitable problem of radiotherapy. Multiple experiments demonstrate that hypoxia and radiation resistance are associated [43, 44]. Chang *et al* showed that ^18^F-FMISO PET technology was suitable for dose painting in HNSCC [45]. This radiotherapy approach not only increased tumor control probability, but also increased uncomplicated tumor control probability. Thus it revealed to be a superior method to traditional dose escalation radiotherapy. The authors forecasted that the clinical application of this method would improve the results of radiotherapy and promote the development of individual doses prescriptions. Furthermore, dose painting will be more robust based on dynamic PET analysis described by Thorwarth [39, 46]. However, Cui *et al* and Ljungkvist *et al* reported that hypoxic cancer cells *in vivo* and *in vitro* culture had different lifespan, and the turnover of hypoxic tumor cells may relate to the therapeutic response and outcome in patients [7, 47]. That may indicate dose painting would be unhelpful. So, there need to do more experiments to verify the usefulness of dose painting with ^18^F-FMISO PET technology.

### Guiding hypoxic targeted therapy

At present, there has developed a series of hypoxia targeted drugs such as Evofosfamide, Banoxantrone and tirapazamine. They belong to bioreductive prodrugs and are reduced by intracellular oxidoreductases in an oxygensensitive manner to form cytotoxins in order to disrupt the DNA replication fork [48]. Stokes *et al* showed that C6 tumors exhibited more hypoxia and were less perfused than 9L tumors by using ^18^F-FMISO imaging [49]. On the basis of these differences in their tumor hypoxic burden, treatment with Evofosfamide resulted in 4 and 2 folds decreases in tumor growth rates of C6 and 9L tumors, respectively. This had implications for improved patient selection in clinical trials. Rischin *et al* provided the first clinical evidence that hypoxia on ^18^F-FMISO imaging, in patients receiving a nontirapazamine-containing chemoradiotherapy regimen, was associated with a high risk of locoregional failure [50].

Several of hypoxia targeted drugs has been used in clinical studies, especially in larger primary tumors. But most of them, such as Evofosfamide failed in phase Ⅲ of soft tissue sarcoma and pancreatic cancer. Primary tumor growth accounts for few cancer-related deaths, for its outcome is necrosis or metastasis. Cancer mortality is mostly due to the development of metastatic disease. Hypoxia is a significant microenvironmental factor to induce invasion and metastasis. It suggests to be a master regulator role in metastasis. For each step of the metastasis process, from the initial epithelial mesenchymal transition to the ultimate organotropic colonization, is associated with hypoxia/HIF-1α-regulated target genes [51, 52]. Metastatic disease especially micrometastasis is highly hypoxia. Thus, in recent years, cancer researches have begun to focus on the changes in the tumor microenvironment of micrometastasis [53]. Huang *et al* compared the uptake of ^18^F-FMISO with the hypoxia-staining marker pimonidazole in subcutaneous xenograft and peritoneal metastases generated by non-small cell lung cancer cell lines (A549 and HTB177), and found significant overlap between the two markers [6]. Micrometastases were too small for detection. In another study, the same authors showed that, in addition to the normal uptake in the liver, bladder and intestines, ^18^F-FMISO accumulated strongly in the left peritoneal wall in a A549 peritoneal micrometastases model [54]. Hematoxylin and eosin staining confirmed that this region of severe hypoxia corresponded to small metastases. Furthermore, positive pimonidazole staining in ascites cells collected from the ascites fluid of the peritoneal cavity further demonstrated that ^18^F-FMISO PET could detect small metastases. Therefore, hypoxic targeted therapy may be more effective in metastasis with the help of ^18^F-FMISO PET.

### Management of therapeutic efficacy

^18^F-FMISO PET is suitable for management of therapeutic efficacy, as it can reflect the degree of cell reoxidation. For instance, by comparing ^18^F-FMISO uptake before and after radiotherapy with chemotherapy, it was possible to determine the best conditions for killing cancer cells with these treatments [55]. Furthermore, Spence *et al* showed that the volume of the hypoxic tumor and the maximum level of hypoxia assessed by ^18^F-FMISO PET before radiotherapy, correlated negatively with the progression time and survival [22]. Thus, this imaging technology is a promising new approach for clinical diagnosis, prognosis estimation and evaluation of therapy efficacy. About 70% of breast cancers belong to the hormone-dependent type, which is characterized by the overexpression of hormone receptors [56]. Although endocrine therapy is an effective treatment for positive estrogen receptor breast cancer, about 30% of these tumors develop either primary or acquired resistance [56–58]. To assess the feasibility of using ^18^F-FMISO to monitor therapy resistance, Cheng *et al* compared PET/CT scans of positive estrogen receptor patients performed before and after treatment. ^18^F-FDG uptake showed no significant correlation with the results of the treatment, but a positive correlation was found with ^18^F-FMISO uptake. Furthermore, when tumor/blood ratio ≥ 1.2, ^18^F-FMISO uptake was a predictor of endocrine resistance in 88% of the tumors [56]. Thus, ^18^F-FMISO imaging could significantly improve breast cancer treatment. To investigate the possible prognostic value of initial tumor hypoxia or its changes under sunitinib therapy, Hugonnet *et al* evaluated initial tumor hypoxia in metastatic renal cell carcinoma and its changes after sunitinib treatment using ^18^F-FMISO PET/CT [59]. They found patients with initially hypoxic targets had shorter progression-free survival than others. But the prognostic value of ^18^F-FMISO PET/CT performed earlier after initiation of treatment, with dynamic acquisition, needed to be assessed in further studies.

In all, imaging with ^18^F-FMISO presents many advantages: (i) it is a noninvasive and comprehensive method to assess hypoxia; (ii) ^18^F-FMISO is specifically retained in hypoxia tissues; (iii) it has a good reproducibility; (iv) it can be used for guiding therapy. On the other hand, due to its high radioactive concentration in liver, kidney and intestine, ^18^F-FMISO is not suitable for abdominal tumor imaging. Furthermore, when compared with ^18^F-FDG, ^18^F-FMISO produces images of inferior quality and contrast. Moreover, although ^18^F-FMISO imaging can continuously measure hypoxia, it requires at least one day of interval between scans. It influences monitoring of temporal heterogeneity.

## OTHER HYPOXIA IMAGING AGENTS

To overcome the limitations of ^18^F-FMISO, new researches and improved derivatives are in constant development. For instance, small changes in the structure of ^18^F-FMISO make the hydrophilic ^18^F-FETNIM (^18^F-Fluoroerythronitroimidazole). The main advantage of ^18^F-FETNIM when compared to similar compounds is its higher hydrophilicity and relatively lower neurotoxicity, in addition to a lower peripheral metabolic rate [60] .The radiochemical purity of ^18^F-FETNIM can typically reach 99%. This tracer is also easier to prepare and less costly than ^18^F-FMISO. However, despite these advantages, more research is necessary to determine its clinical prospects [61].

^18^F-FAZA (^18^F-Fluoroazomycinarabinoside) is developed in recent years and currently being tested in hypoxic PET imaging studies. It is thought that ^18^F-FAZA accumulates in hypoxic cells through a similar mechanism as imidazole derivatives. ^18^F-FAZA has stronger affinity to the tumor hypoxic areas than other tracers and it is removed faster in blood and muscle tissue [62]. The typical radiochemical yielding of this tracer is 20.7 ± 3.5% at the end of the reaction [63]. Furthermore, the precursor can be labeled in various manners, including by iodinated counterparts (^123^I/^124^I-IAZA) [64].

^18^F-HX_4_ (^18^F-3-fluoro-2-(4-((2-nitro-1H-imidazol-1-yl)methyl)-1H-1, 2, 3-triazol-1-yl)-propan-1-ol) has higher sensitivity, specificity, faster clearance, and shorter injection-imaging time compared with ^18^F-FMISO in a pilot clinical study of 12 patients with head and neck cancer [65]. Biodistribution and dosimetry studies in healthy monkeys and humans showed that the highest up take of ^18^F-HX_4_ was found in the bladder, kidney, liver and gastrointestinal tract uptake was relatively low [66]. Repeated hypoxia PET scans with ^18^F-HX_4_ provided reproducible and spatially stable results in patients with head and neck cancer and patients with lung cancer [67]. ^18^F-HX_4_ PET imaging can be used to assess the hypoxic status of tumors and has the potential to aid hypoxia-targeted treatments.

^18^F-EF series is mainly included EF_1_, EF_3_,and EF_5_ which are from etanidazoles compounds. The combination with hypoxic cells is more specific, while the toxicity decreases significantly. However, in a comparative PET imaging study, no superiority of ^18^F-EF_3_ (^18^F-(2-2-nitroimidazol-1-yl)-*N*-(3,3,3-trifluoropropyl)-acetamide) to ^18^F-FMISO for the evaluation of hypoxia [68]. Other chemical class of hypoxia radiotracer currently under scrutiny in preclinical and clinical studies is typically labeled by ^60^Cu, ^61^Cu, ^62^Cu, and ^64^Cu, for example, ^64^Cu-ATSM (^64^Cu- diacetyl-bis (N^4^-methylthiosemicarbazone)). This compound shows high variability in tumor uptake between different tumor types *in vitro* [69]. However, ^64^Cu-ATSM is not valid in all types of tumor such as fibrosarcoma. Further studies are needed to define retention mechanisms for this PET marker [70].

## CONCLUSIONS

This review begins by explaining the concept of hypoxia and introducing the most widely used hypoxic imaging agent: ^18^F-FMISO. A comprehensive discussion of ^18^F-FMISO applications for hypoxia imaging is presented. ^18^F-FMISO shows great promise as a clinical hypoxia-imaging agent, however, it also presents certain limitations. Obviously, a method is difficult to meet all the needs of hypoxia imaging. Therefore, it need a combination of multiple imaging methods to explore tumor hypoxia in deeply. Future studies should therefore focus on improving the properties of ^18^F-FMISO and trying to combine this tracer with other hypoxia imaging methods in order to increase sensitivity and specificity of hypoxia detection. Thus, ^18^F-FMISO imaging currently offers vast potential for development to ultimately provide a comprehensive description of tumor hypoxic states and hence better guide clinical assessment and therapy design.

## Abbreviations

^18^F-FMISO PET: ^18^F-Fluoromisonidazole positron emission tomography
PO_2_: oxygen partial pressure
HIF-1: hypoxia inducible factor 1
VEGF: vascular endothelial growth factor
SUV_max_: maximum standardized uptake value
BOLD fMRI: blood oxygenation-level dependent functional magnetic resonance imaging
HNSCC: head and neck squamous cell carcinoma
RCC: renal cell carcinoma
^18^F-FETNIM: ^18^F-Fluoroerythronitroimidazole
^18^F-FAZA: ^18^F-Fluoroazomycinarabinoside
^18^F-HX_4_: ^18^F-3-fluoro-2-(4-((2-nitro-1H-imidazol-1-yl)methyl)-1H-1, 2, 3-triazol-1-yl)-propan-1-ol
^18^F-EF_3_: ^18^F-(2-2-nitroimidazol-1-yl)-*N*-(3,3,3-trifluoropropyl)-acetamide
^64^Cu-ATSM: ^64^Cu-diacetyl-bis (N^4^-methylthiosemicarbazone)
